# Prognostic value of red blood cell distribution width in patients with acute pulmonary embolism: A protocol for systematic review and meta-analysis

**DOI:** 10.1097/MD.0000000000025571

**Published:** 2021-05-07

**Authors:** Ye Liao, Chunsheng Yang, Banu Bakeer

**Affiliations:** a Medical Intensive Care Unit, West China Hospital, Sichuan University, Chengdu, Sichuan; b Department of Rehabilitation Medicine, The First Affiliation Hospital of Xinjiang Medical University, Urumqi, PR China.

**Keywords:** mortality, prognosis, pulmonary embolism, red blood cell distribution width

## Abstract

**Background:**

Prior reports have suggested that the red blood cell distribution width (RDW) parameter could be measured as a prognostic indicator in pulmonary embolism (PE) patients, thereby helping to guide their care. However, no systematic analyses on this topic have been completed to date, and the exact relationship between RDW and PE remains to be fully clarified. We will therefore conduct a systematic literature review with the goal of defining the correlation between RDW and mortality in acute PE cases.

**Methods:**

The EMBASE, Web of Knowledge, PubMed, ClinicalTrials.gov, and Cochrane Library databases will be searched for all relevant studies published from inception through March 2021 using the following search strategy: (“red blood cell distribution width”) AND (“pulmonary embolism”). Two authors will independently identify eligible studies and extract data. The *Q* and *I*^2^ statistics will be used to judge heterogeneity among studies.

**Results:**

This study will establish the relative efficacy of RDW as a metric for predicting PE patient mortality.

**Conclusions:**

This study will offer a reliable, evidence-based foundation for the clinical utilization of RDW as a tool for gauging mortality risk in acute PE patients.

**Ethics and dissemination:**

As this is a protocol for a systematic review of previously published data, no ethical approval is required. Electronic dissemination of study results will be done through a peer-review publication or represented at a related conference.

## 1 Introduction

Acute pulmonary embolism (PE) is an extremely dangerous medical condition with high mortality rates that is typically secondary to the incidence of venous thromboembolism (VTE). Acute PE is the third major reason of death in the United States of America,^[1]^ and contributes significantly to the global cardiovascular disease burden with respect to associated morbidity, mortality, and healthcare costs.^[2,3]^ It is thus important that clinicians be able to rapidly identify cases of serious PE in order to provide patients with more aggressive care that has the potential to improve prognosis.

Red blood cell (RBC) distribution width (RDW) is a clinical indicator of pro-thrombotic status that measures RBC sizing variability based on mean corpuscular volume (MCV). This hematological parameter can be readily, safely, and inexpensively measured using an automated flow cytometer as part of a complete blood count.^[4]^ In prior studies, RDW values have been reported to correlate with acute PE patient mortality risk.^[5–7]^ Higher RDW levels may be indicative of a range of pathological processes including inflammatory stress, and may be related to poor outcomes in PE patients,^[8]^ although this conclusion is controversial.

To evaluate the relationship between RDW at admission and early mortality in acute PE patients, a meta-analysis and systematic review will be conducted.

## 2 Methods and analysis

### 2.1 Registration

This meta-analysis protocol is based on the Preferred Reporting Items for Systematic Reviews and meta-analysis Protocols (PRISMA-P) statement guidelines. The PRISMA-P checklist for the protocol is provided in the PRISMAP-checklist. This protocol has been registered on International Prospective Register of Systematic Reviews database. The registration number was INPLASY202130036.

### 2.2 Eligibility criteria

To be eligible for inclusion in this study, articles must include the criteria given below: studies with diagnosed PE patients ≥18 years old and a minimum hospitalization duration of 24 hours; conference abstracts are only eligible for inclusion when they provide sufficient information to permit associated analyses; studies utilizing RDW as a metric to predict PE patient mortality. Studies will be excluded from this analysis if they include patients <18 years old, patients with cancers, or patients with incomplete data.

### 2.3 Search strategy

**Figure 1:**
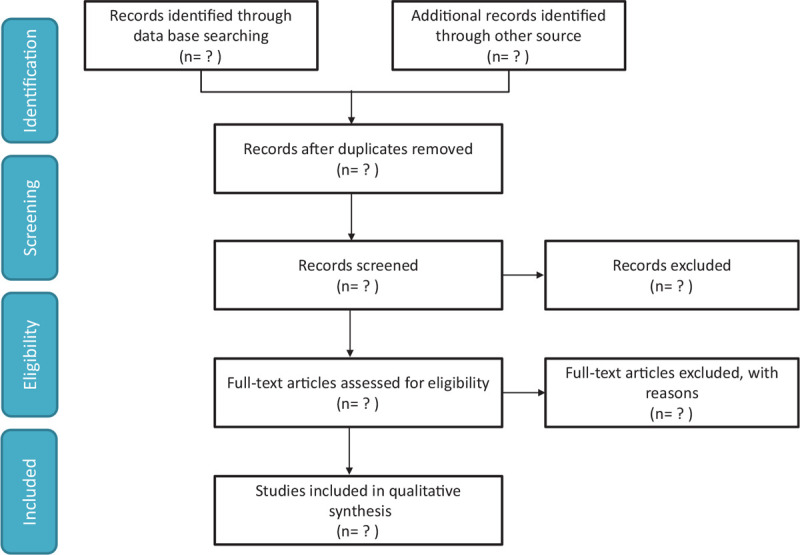
A flowchart outlining the study selection process for the proposed meta-analysis.

The EMBASE, Web of Knowledge, PubMed, ClinicalTrials.gov, and Cochrane Library databases will be searched without any language restrictions for relevant studies published from inception through March 2021with the following search strategy: (“red blood cell distribution width”) AND (pulmonary embolism). The citations of relevant studies and other pertinent primary and review articles will be explored to specify other potentially related investigations. Authors of meeting abstracts will be searched in the PubMed database to identify full-length articles where possible. To reduce the risk of publication bias, our comprehensive analysis will include both published and unpublished studies where possible (Fig. 1).

### 2.4 Data extraction and risk of bias assessment

The exploration strategy detailed above will be executed by 2 researchers independently of one another, and study quality will be assessed using the QUADAS-2 checklist^[9]^ and the Newcastle-Ottawa Quality Assessment Scale.^[10]^ Any inconsistencies among these 2 scientists will be resolved through discussion with a third investigator. A standardized form will be used to extract key data from each investigation including first author name, country of publication, year of publication, investigation type, study population, sample size, PE definition criteria, outcomes studied (mortality rate), and red blood cell distribution width level. The Newcastle-Ottawa Scale (NOS) will be utilized to rate individual investigation internal validity, and the risk of publication bias will be evaluated utilizing funnel plots.

### 2.5 Statistical analysis

The Review Manager computer software (RevMan v5.3; Cochrane Collaboration) will be used for all pairwise comparisons. The *Q* and *I*^2^ statistics will be used to judge potential heterogeneity among studies, and reporting bias will be detected by visually inspecting funnel plots. *P* < .05 will be the significance threshold for these analyses.

## 3 Discussion

Pulmonary embolism patients face high rates of morbidity and mortality, and PE is thus a major contributor to the overall global disease burden.^[11,12]^ Computed tomography pulmonary angiography (CTPA) is currently the standard approach to evaluating patients suffering from suspected PE.^[12]^ Unlike CTPA, however, RDW can be readily measured by analyzing blood samples obtained through routine peripheral venipuncture.

As RDW measurements are easier to implement in a clinical setting than CTPA or other diagnostic modalities, they have the potential to facilitate rapid decision-making and to decrease the risk of patient mortality. In the proposed meta-analysis, we will therefore explore the prognostic utility of RDW in acute PE patients. Prior studies have reported a significant relationship between RDW and acute PE incidence, but these findings remain controversial.^[13]^

To establish the relative utility of RDW as a means of predicting PE-related mortality, we will conduct the meta-analysis and systematic review outlined in the present study protocol. The results of our study will be reported in strict compliance with PRISMA criteria, and will add to the current literature by providing compelling evidence with the potential to improve care management for patients in need.

## Acknowledgments

The authors would like to acknowledge the participants and their families for taking part in the study.

## Author contributions

**Conceptualization:** Liao Ye.

**Data curation:** Liao Ye.

**Formal analysis:** Liao Ye, Yang Chun sheng.

**Funding acquisition:** Liao Ye, Yang Chun sheng, Banu Bakeer.

**Investigation:** Liao Ye.

**Methodology:** Liao Ye.

**Project administration:** Liao Ye.

**Resources:** Liao Ye, Yang Chun sheng, Banu Bakeer.

**Software:** Liao Ye.

**Supervision:** Liao Ye, Yang Chun sheng, Banu Bakeer.

**Validation:** Liao Ye, Yang Chun sheng, Banu Bakeer.

**Visualization:** Liao Ye, Yang Chun sheng, Banu Bakeer.

**Writing – original draft:** Liao Ye.

**Writing – review & editing:** Liao Ye.
